# Intravenous Thrombolysis Improved Aphemia and Confirmed the Dominant Precentral Gyrus as the Responsible Lesion

**DOI:** 10.7759/cureus.46964

**Published:** 2023-10-13

**Authors:** Tsuyoshi Tsukada, Michiya Kubo, Soshi Okamoto, Masato Hirao, Yukio Horie

**Affiliations:** 1 Neurological Surgery, Saiseikai Toyama Hospital, Toyama, JPN; 2 Neurology, Saiseikai Toyama Hospital, Toyama, JPN

**Keywords:** lesion studies, intravenous thrombolysis, ischemic stroke, dominant precentral gyrus, aphemia

## Abstract

Aphemia is now considered an impairment of speech production. We present a case of an 89-year-old right-handed woman who received intravenous thrombolysis with a recombinant tissue plasminogen activator for the ischemic symptom “loss of speech” and recovered with an ischemic lesion of the left precentral gyrus. The patient had untreated atrial fibrillation. Neurological examination showed that her level of consciousness was alert, with normal comprehension and mild lower facial droop. Head computed tomography (CT) did not reveal a hemorrhagic lesion. To treat the acute ischemic stroke, she received a recombinant tissue plasminogen activator. Just after thrombolysis, she started to speak. Then, magnetic resonance imaging (MRI) revealed an acute ischemic infarction in the dominant precentral gyrus. Follow-up MRI revealed the peripheral middle cerebral artery territory infarction in the left precentral gyrus, but she still could speak. The symptom of “loss of speech” was considered aphemia. By intravenous thrombolysis, impaired speech production in our patient was believed to be caused by an infarction in the dominant precentral gyrus.

This case also demonstrated that the rare clinical symptom was due to an ischemic stroke in the territory of the distal middle cerebral artery. Clinicians who engage in stroke care need to know the rare symptoms of aphemia in the era when mechanical thrombectomy could be considered a promising treatment option for distal medium vessel occlusion.

## Introduction

Aphemia or inhibition of speech is now considered an akinetic motor sign of speech production [1]. Several case reports and studies have shown that the dominant precentral gyrus is related to the area of the brain with speech impairment such as aphemia or speech apraxia [2-8]. On the other hand, injury in the area involving the dominant inferior frontal lobe or so-called Broca area leads to non-fluent Broca aphasia which is the most important differential diagnosis of aphemia. The classical study of the lesion and the mapping of the emerging lesion network to elucidate the functional area of the brain are complementary approaches [9]. This is because lesion network mapping uses patient intrinsic brain imaging and networks derived from healthy volunteers. If lesions have a compensatory effect on their network, this means that lesion network mapping not necessarily leads to correct findings. To overcome this disadvantage, a classical lesion study has a role. In the neurosurgical area, brain mapping by direct electrocortical stimulation has played a role in elucidating brain function, and the study of aphemia is no exception [10]. Considering the compensatory effect on the damaged regions of the brain, reporting a case during the acute stage of stroke is valuable to confirm previously suggested findings of brain function. We present the case of a patient with “loss of speech” with ischemic stroke in the dominant precentral gyrus that was recovered by intravenous thrombolysis. Although previous studies have suggested that the dominant precentral gyrus is related to aphemia in stroke and brain tumor patients [2-8], our presented case confirmed the finding and causal relationship between the lesion and symptom.

## Case presentation

**Figure 1:**
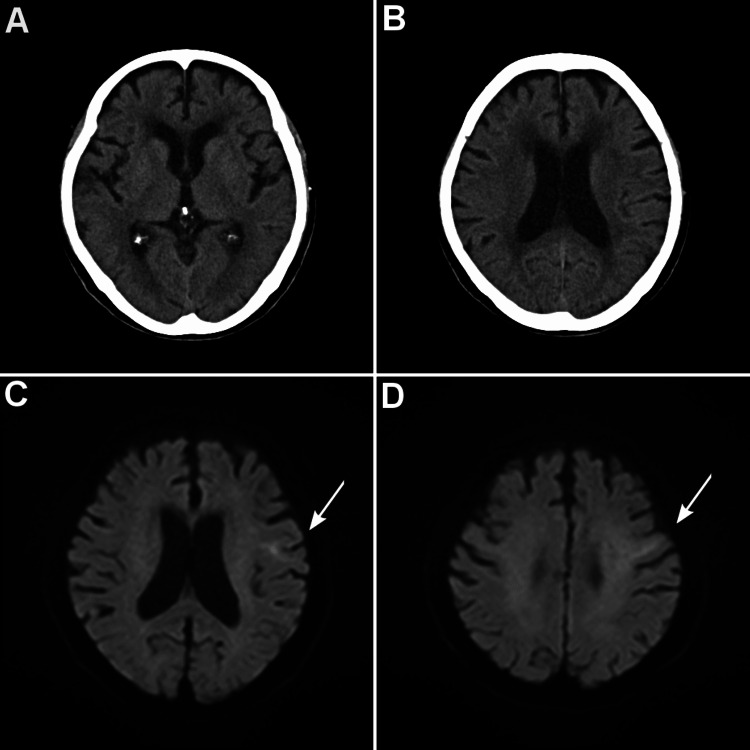
Initial Head CT and MRI just after intravenous thrombolysis, demonstrating an infarct in the left precentral gyrus. Head CT scan (A, B). There were no apparent early ischemic lesions—axial diffusion weight imaging (DWI) (C, D). The arrow indicates an infarction.

An 89-year-old right-handed woman with untreated atrial fibrillation (AF) and acute onset of loss of speech and inability to swallow came to the emergency department with her family. Neurological examinations demonstrated mild lower facial droop and that her consciousness was alert. No apparent hemiparesis was observed. However, she could communicate with her gestures and had normal verbal comprehension. Head computed tomography (CT) scanning did not reveal a hemorrhagic injury (Figure 1).

**Figure 2:**
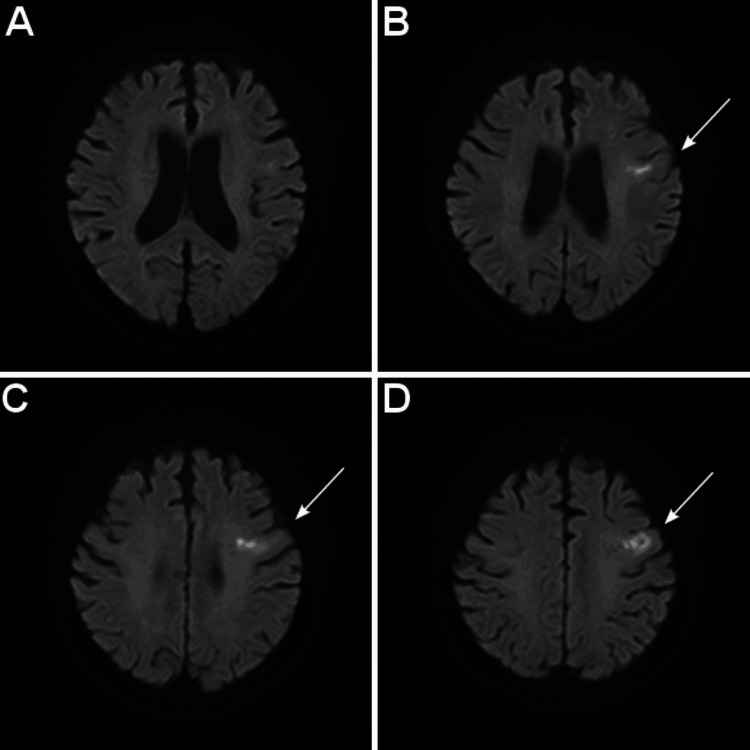
MRI a week after intravenous thrombolysis, revealing the peripheral middle cerebral artery territory infarction in the left precentral gyrus. Axial DWI (A–D). The arrow indicates an infarction. Note the sparing cortex of the left precentral gyrus.

Blood work showed a mild elevated D-dimer level of 1.1 µg/mL, but other blood indicators were within the normal range. As an acute ischemic stroke, she received recombinant tissue plasminogen activator (tPA) therapy (0.6 mg/kg) four hours after onset. Within several minutes after thrombolysis, she regained the ability to say her name and recovered her mild lower facial droop. Then, a first-time MRI revealed an acute ischemic infarction in the left precentral gyrus (Figure 1). The stroke was thought to be cardioembolism and was treated with a heparin drip, followed by switching to edoxaban (30 mg once daily). A week later, a follow-up MRI revealed the peripheral middle cerebral artery territory infarction in the left precentral gyrus (Figure 2).

There was no history of recurrent ischemic lesions. She still could speak with slight difficulty in finding words and the feature of speech was non-fluent. Although we could not exclude the possibility of coexisting aphasia because of the missing writing examination, the “loss of speech” was likely to be aphemia, apraxia of speech from the location of the ischemic lesion that did not include Broca’s area (Figures 1, 2). The patient was hospitalized for about a month. Despite the rehabilitation from the early stage of the stroke, the patient had difficulty in finding words in spontaneous speech, and the amount of speech was limited. During her hospitalization, pacemaker implantation was performed for sick sinus syndrome that coexisted with AF three weeks after stroke. The patient was discharged to a rehabilitation hospital with a modified Rankin scale of 1 with no significant disability despite impairment of speech.

## Discussion

**Figure 3:**
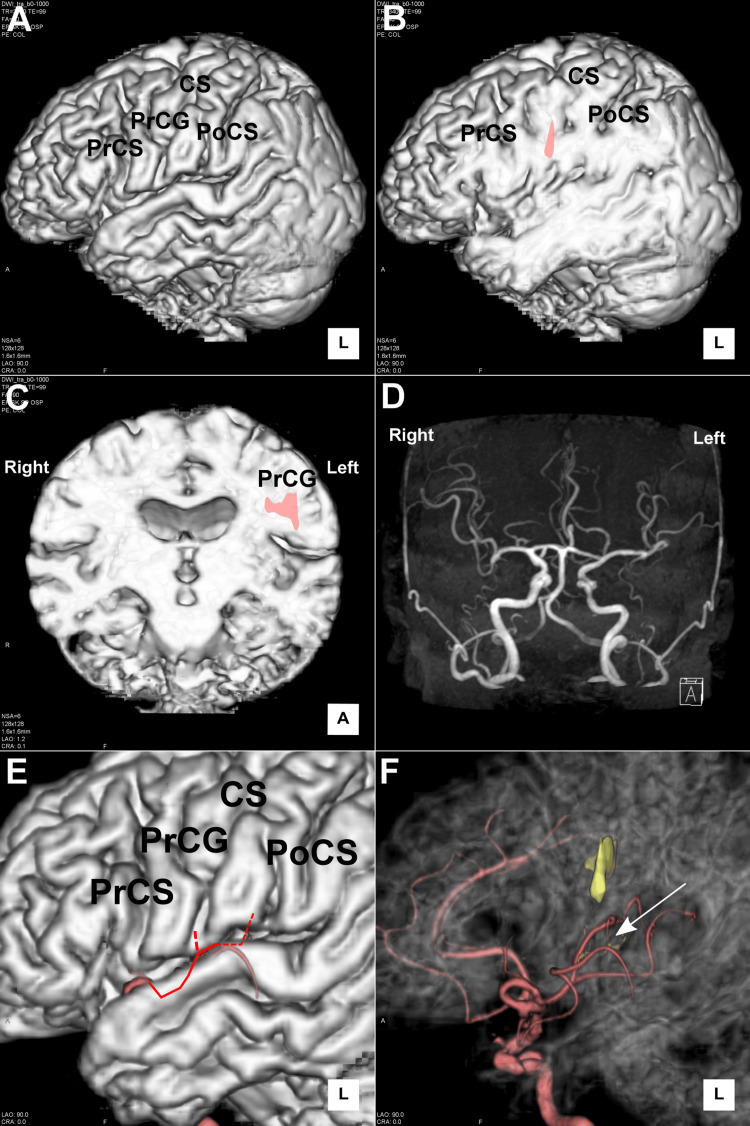
Lesion and middle cerebral artery overlaid on the MRI surface (after thrombolysis). Infarct overlaid on the MRI surface rendering (A, B). Sagittal section at the infarction (B). Coronal section of the MRI surface rendering (C). The pink lesion represents the infarction in the precentral gyrus. Magnetic resonance angiography (MRA) (D). MRA overlaid on the MRI surface rendering (E), transparent image (F). The red line indicates the superior trunk of the middle cerebral artery from which the central artery originates. The red dotted line indicates a double cortical artery of the central artery. Note the configuration of the opercular portion of the middle cerebral artery, which is thought to be the central artery (shown in a red dotted line). CS: Central sulcus; PrCG: precentral gyrus; PrCS: precentral sulcus; PoCS: postcentral sulcus.

Aphemia or inhibition of speech is defined as impairment of speech production and now differentiated from aphasia in terms of symptoms and related areas of the brain [1,5]. To date, studies have been conducted on lesions, and the attributed lesion associated with aphemia was almost consistently on the dominant side of the precentral gyrus [2-7]. This case demonstrates that the lesion accounts for aphemia; “loss of speech” was attributed to the dominant left precentral gyrus by analyzing serial magnetic resonance findings after intravenous thrombolysis in the hyperacute stage of stroke. First of all, it should be noted that we confirmed the causal relationship between stroke in the dominant precentral gyrus and aphemia by achieving the resolution of the symptom with intravenous thrombolysis. Second, this case reports in the hyperacute stage of stroke thereby removing the effect of neural plasticity or compensatory effect which are a matter of previous lesion studies [9]. Therefore, we precisely determined that the dominant precentral gyrus is the responsible lesion as aphemia. From the ischemic lesions demonstrated on serial MRI performed after thrombolysis, we speculated that the lower to mid-left precentral gyrus was responsible for the “loss of speech.” As responsible lesions for “loss of speech,” the dominant posterior inferior frontal area, operculum, and precentral gyrus have been proposed and have gained consensus from lesion studies. Furthermore, electrocortical stimulation of the precentral gyrus has been confirmed to play a major role in the motor programming of speech production [10]. In our case, the lower to mid-precentral gyrus was considered the ischemia lesion responsible for the symptom of “loss of speech,” and the occlusive portion was the left opercular portion of the middle cerebral artery suggested by magnetic resonance angiography (Figure 3).

This occlusive artery was considered a central artery because of the perfusion territory and its configuration [11]. The implication of these findings for clinical practice is two-fold. First, as Stachyra et al. stated, the differential diagnosis should include aphemia due to ischemic stroke of the dominant inferior frontal gyrus [12]. Second, thrombectomy and intravenous thrombolysis for distal medium vessel occlusion (DMVO) have become important procedures in stroke care [13]. Therefore, we need to know the stroke symptoms due to occlusion of DMVO and properly select the appropriate treatment for each case of DMVO in terms of treatment-related complications.

## Conclusions

Intravenous thrombolysis with tPA allowed the functional evaluation of specific symptoms, namely, “loss of speech,” an aphemia that reflects a brain injury due to ischemic stroke. Stroke of the dominant inferior frontal gyrus should be considered when seeing a patient presenting with a sudden onset of aphemia. Our case confirmed that the dominant precentral gyrus had a role in speech production in a case of acute stage stroke.
